# Genomic analysis of *Vibrio cholerae* O1 isolates from cholera cases, Europe, 2022

**DOI:** 10.2807/1560-7917.ES.2024.29.36.2400069

**Published:** 2024-09-05

**Authors:** Caroline Rouard, David R Greig, Thamida Tauhid, Susann Dupke, Elisabeth Njamkepo, Ettore Amato, Boas van der Putten, Umaer Naseer, Marion Blaschitz, Georgia D Mandilara, James Cohen Stuart, Alexander Indra, Harold Noël, Theologia Sideroglou, Florian Heger, Maaike van den Beld, Astrid Louise Wester, Marie-Laure Quilici, Holger C Scholz, Inga Fröding, Claire Jenkins, François-Xavier Weill

**Affiliations:** 1 Institut Pasteur, Université Paris Cité, Unité des Bactéries pathogènes entériques, Centre National de Référence des Vibrions et du choléra, Paris, France; 2 Gastrointestinal Bacteria Reference Unit (GBRU), UK Health Security Agency, London, United Kingdom; 3 Public Health Agency of Sweden, Unit for Laboratory Surveillance of Bacterial Pathogens, Solna, Sweden; 4 Centre for Biological Threats and Special Pathogens (ZBS 2), National Consultant Laboratory for Human Pathogenic *Vibrio* species, Robert Koch Institute, Berlin, Germany; 5 Norwegian Institute of Public Health, Department of Infection Control and Preparedness, Oslo, Norway; 6 Centre for Infectious Disease Control, Institute for Public Health and the Environment (RIVM), Bilthoven, The Netherlands; 7 Austrian Agency for Health and Food Safety, Vienna, Austria; 8 National Reference Centre for *Salmonella*, School of Public Health, University of West Attica, Athens, Greece; 9 Department of Medical Microbiology, Noordwest Ziekenhuisgroep, Alkmaar, The Netherlands; 10 Paracelsus Medical University of Salzburg, Salzburg, Austria; 11 Santé publique France, Saint Maurice, France; 12 Directorate of Epidemiological Surveillance and Intervention for Infectious Diseases, National Public Health Organization (NPHO), Athens, Greece; 13 Norwegian Institute of Public Health, Department of Bacteriology, Norwegian Reference Laboratory for Cholera, Oslo, Norway

**Keywords:** Cholera, Vibrio cholerae, Europe, 2022, whole-genome sequencing, microbial genomics, surveillance

## Abstract

**Background:**

The number of cholera cases reported to the World Health Organization (WHO) in 2022 was more than double that of 2021. Nine countries of the WHO European Region reported 51 cases of cholera in 2022 vs five reported cases in 2021.

**Aim:**

We aimed to confirm that the *Vibrio cholerae* O1 isolates reported by WHO European Region countries in 2022 belonged to the seventh pandemic El Tor lineage (7PET). We also studied their virulence, antimicrobial resistance (AMR) determinants and phylogenetic relationships.

**Methods:**

We used microbial genomics to study the 49 *V. cholerae* O1 isolates recovered from the 51 European cases. We also used > 1,450 publicly available 7PET genomes to provide a global phylogenetic context for these 49 isolates.

**Results:**

All 46 good-quality genomes obtained belonged to the 7PET lineage. All but two isolates belonged to genomic Wave 3 and were grouped within three sub-lineages, one of which, Pre-AFR15, predominated (34/44). This sub-lineage, corresponding to isolates from several countries in Southern Asia, the Middle East and Eastern or Southern Africa, was probably a major contributor to the global upsurge of cholera cases in 2022. No unusual AMR profiles were inferred from analysis of the AMR gene content of the 46 genomes.

**Conclusion:**

Reference laboratories in high-income countries should use whole genome sequencing to assign *V. cholerae* O1 isolates formally to the 7PET or non-epidemic lineages. Periodic collaborative genomic studies based on isolates from travellers can provide useful information on the circulating strains and their evolution, particularly as concerns AMR.

**Key public health message**

**What did you want to address in this study and why?**

Cholera is an acute diarrhoeal infection caused by the bacterium *Vibrio cholerae*. The disease is most common in African and Asian countries. The number of cholera cases more than doubled worldwide between 2021 and 2022. In Europe, 51 cases, mostly infected abroad, were reported in 2022 vs only five cases in 2021. We studied the European *Vibrio cholerae* O1 isolates to gain information about them and their susceptibility to antimicrobials.

**What have we learnt from this study?**

Most of these isolates belonged to a strain that emerged recently and has caused cholera outbreaks in Asia and Africa, probably making a major contribution to the global upsurge of cholera cases in 2022. Fortunately, all these isolates were susceptible to most of the recommended antimicrobials for cholera treatment.

**What are the implications of your findings for public health?**

In high-income countries, we recommend surveillance by whole genome sequencing, which allows the formal identification of this bacterium and robust tracking of the various strains circulating worldwide.

## Introduction

Cholera is an acute diarrhoeal disease caused by ingestion of the bacterium *Vibrio cholerae.* Only two serogroups of this species are associated with cholera: O1, and more rarely, O139, when strains of these serogroups produce the cholera toxin encoded by the CTX prophage [1]. This toxin causes the symptoms of the disease, which range from mild to severe acute watery diarrhoea and can lead to death within hours if untreated. Treatment is based principally on oral rehydration, with the administration of intravenous fluids and antimicrobials in severe cases [1]. The bacterium is transmitted through direct interhuman contact and indirectly via water and food contaminated with faecal material. Cholera prevention is, thus, based on improvements to hygiene, sanitation and access to safe water.

**Figure 1:**
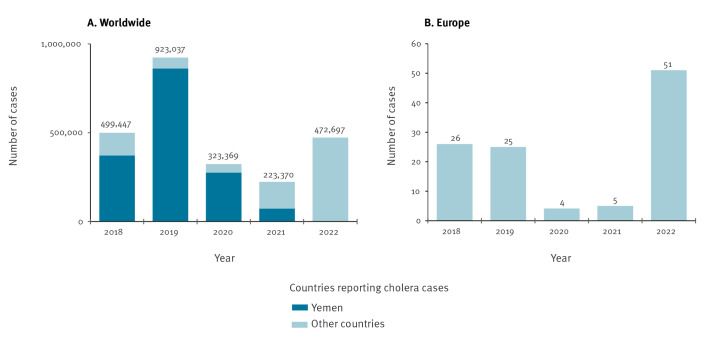
Total number of cholera cases reported to the World Health Organization (WHO) (n = 2,441,920) and cholera cases reported by European countries (n = 111), 2018–2022

The current cholera pandemic, the seventh, began in 1961 and is caused by *V. cholerae* O1 of the El Tor biotype. It began in Sulawesi (Indonesia) and spread across Asia to Southern Asia, which remains an important cholera hotspot, with India and Bangladesh particularly affected [2]. It has also affected many countries in Africa since 1970 and in Latin America since 1991 [3,4]. However, the two largest cholera outbreaks in modern history have occurred in recent years. The first affected Haiti, with more than 800,000 suspected cases, between 2010 and 2019. After 3 years with no cases, cholera recurred in Haiti in September 2022, with ca 12,000 suspected cases reached by the end of November [5,6]. The second outbreak affected Yemen; with more than 2 million suspected cases since 2016, this outbreak accounts for most of the cholera cases reported worldwide to the World Health Organization (WHO) between 2016 and 2020 (Figure 1A) [7].

Panel A. Countries reporting cholera cases: 2018 (n = 34), 2019 (n = 31), 2020 (n = 27), 2021 (n = 35) and 2022 (n = 44). Cases reported by Yemen are indicated in deep blue and those from other countries in pale blue.

Panel B. Countries reporting cholera cases: 2018 (n = 5), 2019 (n = 6), 2020 (n = 2), 2021 (n = 2) and 2022 (n = 9). In 2022, countries reporting cholera cases were: the United Kingdom (n = 23), France (n = 7), Sweden (n = 7), Germany (n = 6), Norway (n = 3), the Netherlands (n = 2), Austria (n = 1), Greece (n = 1), Italy (n = 1). In 2018, 2019 and 2021, the WHO European Region countries did not report any cholera cases to the WHO. The data for these years were obtained from the European Centre for Disease Prevention and Control (ECDC) (https://www.ecdc.europa.eu/en/cholera/surveillance-and-disease-data) and, after the United Kingdom (UK) left the European Union (EU) in 2020, from the UK Health Security Agency (UKHSA) (personal communication C. Jenkins, January 2024). In 2020, we grouped the two cases reported to the WHO by the Netherlands with the two cases identified in the UK (personal communication C. Jenkins, January 2024).

The bacterium responsible for this pandemic belongs to the seventh pandemic *V. cholerae* El Tor (7PET) lineage [4]. Three genomic groups, Waves 1, 2 and 3, have been described within this lineage [8]. Different sub-lineages have been described within these waves to identify the numerous introductions of the 7PET lineage into Europe, Africa and Latin America from South Asia since 1961 [9-11].

In 2022, the WHO reported an upsurge in cholera cases that had begun in 2021 and an increase in the number of countries reporting cases, including reports of very large outbreaks (> 10,000 suspected cases) from seven countries on two continents (Afghanistan, Cameroon, Democratic Republic of the Congo, Malawi, Nigeria, Somalia and Syria) (Figure 1A) [3]. Moreover, these outbreaks were more deadly, with an increase in case fatality rates. These findings led the WHO to revise the overall cholera risk upwards to very high [12]. In 2022, 51 cholera cases (47 of which were imported cases in travellers, the other four being unrelated to travel), all non-fatal, were reported by the European countries (vs only five cases in 2021). The nine countries reporting cases were the UK (n = 23), France (n = 7), Sweden (n = 7), Germany (n = 6), Norway (n = 3), the Netherlands (n = 2), Austria (n = 1), Greece (n = 1) Italy (n = 1). This is the largest number of cholera cases ever reported by the European countries and the largest number of countries reporting cases in Europe since 2018 (Figure 1B).

We applied whole genome sequencing and genomic epidemiology approaches to the *V. cholerae* O1 isolates reported by the European countries in 2022 to confirm that all these isolates belonged to the 7PET lineage and to characterise their virulence and antimicrobial resistance (AMR) determinants. We also determined their phylogenetic relationships by performing a phylogenomic analysis with > 1,450 contextual 7PET genomes. This genomic analysis of isolates from travellers recently infected in countries that do not report cholera cases to the WHO — either through deliberate choice or due to low laboratory capacities — is a valuable source of information for the supranational surveillance of this disease.

## Methods

### Cholera case definition

According to the Global Task Force on Cholera Control (GTFCC) (https://www.gtfcc.org/), in the absence of a probable or confirmed cholera outbreak, the normal situation of European countries since the mid-1990s [11], a suspected cholera case is defined as a person aged ≥ 2 years with acute watery diarrhoea and severe dehydration or who died from acute watery diarrhoea with no other known cause of death [13]. A confirmed cholera case is defined as any person infected with *V. cholerae* O1 or O139, as confirmed by culture (including seroagglutination) or PCR for species (*V. cholerae*) and serogroup (O1/O139) identification [13]. Furthermore, the bacterial isolate should also be shown to be toxigenic (by PCR) in the absence of an established epidemiological link to a confirmed cholera case or source of exposure in another country [13].

### Isolates

We studied 49 *V. cholerae* O1 isolates, including one isolated in Italy and described in a separate paper [14], obtained from the 51 cholera cases reported by European countries in 2022. Two of these cholera cases were diagnosed by non-culture methods and therefore no *V. cholerae* O1 isolate was recovered from them.

### Whole genome sequencing

Whole genome sequencing was performed by the different participating countries, in accordance with their usual practices, except for the isolate from Greece, which was sequenced in France. As the genomic sequences initially produced by Sweden were Ion Torrent (Thermo Fisher Scientific, the United States (US)) sequences, the seven isolates were subsequently resequenced by Novogene (Novogene, Beijing, China) to generate Illumina (Illumina, San Diego, US) short-read sequences.

Genomic DNA was extracted with the Maxwell Cell DNA Purification Kit (Promega, Madison, US) (France and Sweden), the QIAamp DNA Mini Kit (Qiagen, Hilden, Germany) (Germany), the Qiagen DNeasy Blood and Tissue kit (Qiagen) (the Netherlands), the MagNA Pure 96 DNA and Viral NA Small Volume Kit (Roche, Basel, Switzerland) (Norway), the MagAttract HMW DNA kit (Qiagen) (Austria) or the QIAsymphony system (Qiagen) (Italy and UK), in accordance with the manufacturer’s recommendations. The libraries were prepared with the Nextera XT kit (Illumina) (Austria, France, Germany, Italy and UK), the Illumina DNA Prep kit (the Netherlands), the Novogene NGS DNA Library Prep Set (Novogene) (Sweden) or the KAPA HyperPlus kit (Roche) (Norway). Sequencing was performed with the following Illumina platforms: NextSeq 500 (France and the Netherlands), NextSeq 550 (Norway and the Netherlands), NextSeq 1000 (UK), NextSeq 2000 (Austria), NovaSeq 6000 (Sweden), MiSeq (Germany and Italy) or HiSeq 2500 (UK), generating 100–300 bp paired-end reads, yielding a mean of 139-fold coverage (minimum 26-fold, maximum 297-fold).

### Genomic sequence analysis

All reads were filtered with FqCleanER v.213.07 (https://gitlab.pasteur.fr/GIPhy/fqCleanER) to eliminate adaptor sequences and discard low-quality reads with phred scores < 28 and a length < 70 bp [15]. Taxonomic read classification with Kraken v.2.1.1 was used to confirm that sequencing reads originated from *V. cholerae* and not from a contaminant [16]. Only the genomes satisfying the quality control criteria were retained for further analyses. Short reads were assembled with SPAdes v.3.15.5, with default settings in most cases, but with the parameter ‘--phred-offset 33’ for some genomes [17]. The various genetic markers were analysed with the Basic Local Alignment Search Tool (BLAST) v.2.2.26 against reference sequences of the O1 *rfb* gene, *ctxB*, *wbeT*, and the whole locus of VSP-II, as previously described [9].

The presence and type of acquired antimicrobial resistance genes (ARGs) or ARG-containing structures were determined with ResFinder v.4.0.1 (https://cge.food.dtu.dk/services/ResFinder/), BLAST analysis against GI-15, Tn7, and SXT/R391 integrative and conjugative elements, and PlasmidFinder v.2.1.1 (https://cge.food.dtu.dk/services/PlasmidFinder/). The presence of mutations in the genes encoding resistance to quinolones (*gyrA*, *parC*), resistance to nitrofurans (*nfsA*, nitroreductase, VC0715 and VCA0637, dihydropteridine reductase), or restoring susceptibility to polymyxin B (VC1320, *vprA*) were investigated by manual analysis of the sequences assembled de novo with BLAST, as previously described [9,18].

### Additional genomic data

Raw sequence files and assembled genomes from 1,452 7PET strains were downloaded from the European Nucleotide Archive (ENA, https://www.ebi.ac.uk/ena/) or GenBank (https://www.ncbi.nlm.nih.gov/genbank/). These genomes comprised the 1,443 genomes described by Smith et al. [19] in addition to three genomes from the Philippines (from isolates collected in 2011) [20], four genomes from South Korea (2016) [21] and two genomes from Papua New Guinea (2010) [22]. See Supplementary Table 2 for a list of all 1,452 contextual genomes used in this study, with their accession numbers.

### Phylogenetic analyses

The paired-end reads and draft or assembled genomes were mapped onto the reference genome of *V. cholerae* O1 El Tor N16961, also known as A19 (GenBank accession numbers LT907989 and LT907990) with Snippy version 4.6.0/BWA v.0.7.17 (https://github.com/tseemann/snippy). Single nucleotide variants (SNVs) were called with Snippy v.4.6.0/Freebayes v.1.3.2 (https://github.com/tseemann/snippy), under the following constraints: mapping quality of 60, a minimum base quality of 13, a minimum read coverage of 4 and a 75% read concordance at the locus for a variant to be reported. An alignment of core genome SNVs was produced in Snippy for phylogeny inference.

Repetitive (insertion sequences and the TLC-RS1-CTX region) and recombinogenic (VSP-II) regions in the alignment were masked [9]. Putative recombinogenic regions were detected and masked with Gubbins v.3.2.0 [23]. A maximum likelihood (ML) phylogenetic tree was built from an alignment of 10,882 chromosomal SNVs, with RAxML v.8.2.12, under the GTR model with 200 bootstraps [24]. This global tree was rooted on the A6 genome, the earliest and most ancestral 7PET isolate, collected in Indonesia in 1957 [9], and visualised with iTOL v.6 (https://itol.embl.de) [25].

## Results

**Figure 2:**
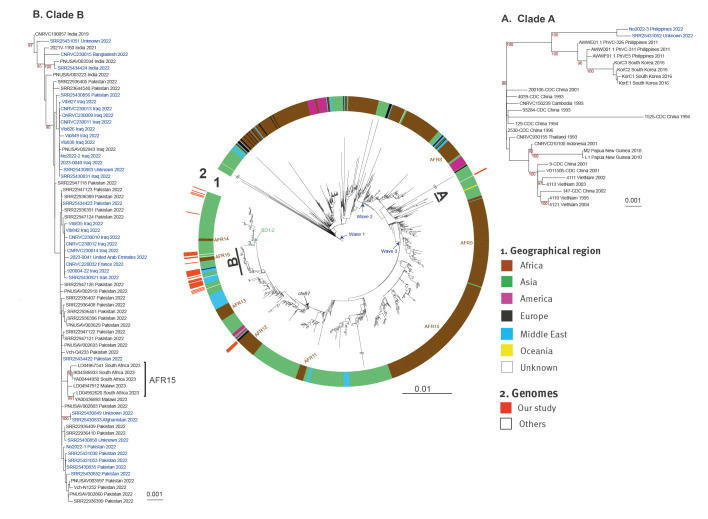
Maximum-likelihood phylogeny of *Vibrio cholerae* O1 El Tor isolates (n = 46), Europe, 2022, compared with reference seventh pandemic *Vibrio cholerae* El Tor genomic sequences (n = 1,452)

In 2022, nine countries of the WHO European Region reported 51 cases of cholera [3] (Figure 1), from which 49 *V. cholerae* O1 isolates were recovered. The genome of the isolate from Italy was already available (SRR23703406) [14], and the remaining 48 isolates were sequenced for this study. In total, 46 of the 49 genomes passed quality control (three genomes were contaminated with other bacterial species and were removed). The basic metadata (country of infection) and genomic characteristics, including the AMR determinants of these 46 isolates, are shown in the Table, see Supplementary Table 1 for further details. We used 1,452 publicly available 7PET genomes to place these 46 *V. cholerae* O1 genomes into the global phylogenetic context of the 7PET lineage. The phylogenetic tree was constructed with 10,882 non-recombinant SNVs from these 1,498 7PET genomes (Figure 2).

**Table:** Main characteristics of *Vibrio cholerae* O1 isolates, Europe, 2022 (n = 46)

Isolate name	Isolation date	Country of infection	Wave	Sub-lineage	AMR determinants
2023–0040	2 Oct^b^	Iraq	3	Pre-AFR15	*dfrA1*, *gyrA*_S83I, *parC*_S85L
2023–0041	23 Nov^b^	UAE	3	Pre-AFR15	*dfrA1*, *gyrA*_S83I, *parC*_S85L
CNRVC230009	14 Jun	Iraq	3	Pre-AFR15	*dfrA1*, *gyrA*_S83I, *parC*_S85L
CNRVC230010	03 Jul	Iraq	3	Pre-AFR15	*dfrA1*, *gyrA*_S83I, *parC*_S85L
CNRVC230011	22 Jul	Iraq	3	Pre-AFR15	*dfrA1*, *gyrA*_S83I, *parC*_S85L
CNRVC230012	22 Jul	Iraq	3	Pre-AFR15	*dfrA1*, *gyrA*_S83I, *parC*_S85L
CNRVC230013	31 Jul	Iraq	3	Pre-AFR15	*dfrA1*, *gyrA*_S83I, *parC*_S85L
CNRVC230014	29 Sep	Iraq	3	Pre-AFR15	*dfrA1*, *gyrA*_S83I, *parC*_S85L
CNRVC230015	1 Nov	Bangladesh	3	Pre-AFR15	*strAB*, *floR*, *sul2*, *dfrA1*, *gyrA*_S83I, *parC*_S85L
22040725-VUM^a^	5 Apr	Bangladesh	3	BD1.2	*strAB*, *floR*, *sul2*, *dfrA1*, *gyrA*_S83I, *parC*_S85L
560/2022	17 Oct	Bangladesh	3	BD1.2	*strAB*, *floR*, *sul2*, *dfrA1*, *gyrA*_S83I, *parC*_S85L
920004–22	29 Sep	Iraq	3	Pre-AFR15	*dfrA1*, *gyrA*_S83I, *parC*_S85L
CNRVC220016	5 Mar	Cameroon	3	AFR12	*dfrA1*, *gyrA*_S83I, *parC*_S85L
CNRVC220032	29 Jun	European country	3	Pre-AFR15	*dfrA1*, *gyrA*_S83I, *parC*_S85L
CNRVC220053	4 Aug	European country^c^	3	AFR12	*dfrA1*, *gyrA*_S83I, *parC*_S85L
CNRVC220058	6 Aug	Cameroon	3	AFR12	*dfrA1*, *gyrA*_S83I, *parC*_S85L
CNRVC220061	6 Aug	European country^c^	3	AFR12	*dfrA1*, *gyrA*_S83I, *parC*_S85L
CNRVC220064	8 Aug	European country^c^	3	AFR12	*dfrA1*, *gyrA*_S83I, *parC*_S85L
SRR25434472	7 Apr	Bangladesh	3	BD1.2	*strAB*, *floR*, *sul2*, *dfrA1*, *gyrA*_S83I, *parC*_S85L
SRR25430923	13 Apr	Bangladesh	3	BD1.2	*strAB*, *floR*, *sul2*, *dfrA1*, *gyrA*_S83I, *parC*_S85L
SRR25434422	14 Apr	Pakistan	3	Pre-AFR15	*strAB*, *floR*, *sul2*, *dfrA1*, *gyrA*_S83I, *parC*_S85L
SRR25434423	20 Apr	Pakistan	3	Pre-AFR15	*dfrA1*, *gyrA*_S83I, *parC*_S85L
SRR25431050	21 Apr	Bangladesh	3	BD1.2	*strAB*, *floR*, *sul2*, *dfrA1*, *gyrA*_S83I, *parC*_S85L
SRR25430852	23 May	Pakistan	3	Pre-AFR15	*strAB*, *floR*, *sul2*, *dfrA1*, *gyrA*_S83I, *parC*_S85L
SRR25430856	17 Jun	Pakistan	3	Pre-AFR15	*dfrA1*, *gyrA*_S83I, *parC*_S85L
SRR25430831	20 Jun	Iraq	3	Pre-AFR15	*dfrA1*, *gyrA*_S83I, *parC*_S85L
SRR25434424	21 Jun	India	3	Pre-AFR15	*strAB*, *floR*, *sul2*, *dfrA1*, *gyrA*_S83I, *parC*_S85L
SRR25430835	17 Aug	Pakistan	3	Pre-AFR15	*strAB*, *floR*, *sul2*, *dfrA1*, *gyrA*_S83I, *parC*_S85L
SRR25430921	19 Aug	Iran	3	Pre-AFR15	*dfrA1*, *gyrA*_S83I, *parC*_S85L
SRR25431038	24 Aug	Pakistan	3	Pre-AFR15	*dfrA1*, *gyrA*_S83I, *parC*_S85L
SRR25430858	30 Aug	Unknown	3	Pre-AFR15	*dfrA1*, *gyrA*_S83I, *parC*_S85L
SRR25430833	1 Sep	Afghanistan	3	Pre-AFR15	*dfrA1*, *gyrA*_S83I, *parC*_S85L
SRR25430849	1 Sep	Unknown	3	Pre-AFR15	*dfrA1*, *gyrA*_S83I, *parC*_S85L
SRR25430883	8 Sep	Unknown	3	Pre-AFR15	*dfrA1*, *gyrA*_S83I, *parC*_S85L
SRR25431053	13 Sep	Pakistan	3	Pre-AFR15	*strAB*, *floR*, *sul2*, *dfrA1*, *gyrA*_S83I, *parC*_S85L
SRR25431052	27 Sep	Unknown	2	None	No AMR genes^d^
SRR25431051	28 Nov	Unknown	3	Pre-AFR15	*strAB*, *floR*, *sul2*, *dfrA1*, *gyrA*_S83I, *parC*_S85L
No2022–1	22 Jun^b^	Pakistan	3	Pre-AFR15	*strAB*, *floR*, *sul2*, *dfrA1*, *gyrA*_S83I, *parC*_S85L
No2022–2	20 Jun^b^	Iraq	3	Pre-AFR15	*dfrA1*, *gyrA*_S83I, *parC*_S85L
No2022–3	18 Dec^b^	Philippines	2	None	No AMR genes^d^
Vib826	23 Jun	Iraq	3	Pre-AFR15	*dfrA1*, *gyrA*_S83I, *parC*_S85L
Vib827	30 Jun	Iraq	3	Pre-AFR15	*dfrA1*, *gyrA*_S83I, *parC*_S85L
Vib835	11 Aug	Iraq	3	Pre-AFR15	*dfrA1*, *gyrA*_S83I, *parC*_S85L
Vib838	2 Sep	Iraq	3	Pre-AFR15	*dfrA1*, *gyrA*_S83I, *parC*_S85L
Vib842	16 Sep	Iraq	3	Pre-AFR15	*dfrA1*, *gyrA*_S83I, *parC*_S85L
Vib849	28 Sep	Iraq	3	Pre-AFR15	*dfrA1*, *gyrA*_S83I, *parC*_S85L

AMR: antimicrobial resistance; UAE: United Arab Emirates.

^a^ Isolate described by Russini et al. 2023 [14].

^b^ Sampling date instead of isolation date.

^c^  Consumption of food imported from Cameroon.

^d^ Only AMR genes encoding resistance to nitrofurans that were present in all 46 isolates.

Further details in Supplementary Table 1.

A6 was used as the outgroup. Blue arrows represent the three genomic waves, the black arrow indicates the acquisition of the *ctxB7* allele and the green arrow indicates the BD1.2 sub-lineage. The colour coding in the inner ring indicates the geographical origins of the isolates and the most recent African sub-lineages (AFR8–AFR15) are shown on the right. The 46 isolates studied here are shown in red in the outer ring. A magnification of two selected clades (A and B) is shown on the right (A) and on the left (B), with blue text indicating the isolates from 2022 studied. For each genome, name or accession number, country in which infection occurred and year of sample collection are indicated at the tips of the tree. Scale bars indicate the number of nt substitutions per variable site. Bootstrap values ≥ 90% are shown in red.

Two isolates, No2022–3 and SRR25431052 (one associated with the Philippines and the other from a patient for whom information about travel was not available), clustered in the Wave 2 group (*ctxB1* allele) (magnification A in Figure 2). These two isolates were grouped together (mean pairwise distance of 11 core genome SNVs) and were phylogenetically close to three isolates collected in the Philippines in 2011 and four isolates collected in South Korea in 2016 [20,21]. No AMR genes were detected in either No2022–3 or SRR25431052. This finding is consistent with observations for the 2011 Philippine and 2016 South Korean isolates, all of which lacked genomic island GI-15 and ICE*Vch*Ban11 (a member of the SXT-R391 SXT family of integrative and conjugative elements (ICE)), chromosomal AMR-encoding elements found in many other closely related Wave 2 isolates [9]. Only resistance to nitrofurans, conferred by point mutations of two genes (VC0715 and VCA0637), was inferred for these two isolates.

The remaining 44 isolates belonged to Wave 3 and clustered with isolates carrying the *ctxB7* allele. They contained the SXT/R391 genomic element ICE*Vch*Ind5, which encodes resistance to streptomycin (*strAB*), sulfonamides (*sul2*), chloramphenicol (*floR*), trimethoprim and the O/129 vibriostatic agent (*dfrA1*), and trimethoprim–sulfamethoxazole (*sul2* and *dfrA1*), or a variant of ICE*Vch*Ind5 with a 10 kb deletion conferring resistance only to trimethoprim and the O/129 vibriostatic agent (*dfrA1*). All 44 isolates carried mutations of the two chromosomal genes (VC0715 and VCA0637) conferring resistance to nitrofurans and point mutations in the quinolone resistance-determining regions (QRDRs) of chromosomal genes *gyrA* (S83I) and *parC* (S85L), resulting in nalidixic acid resistance with decreased susceptibility or even resistance to ciprofloxacin as described in various previous studies [18,19,26,27].

These 44 isolates clustered into three discrete groups. The five isolates with a link to Cameroon clustered within the AFR12 (formerly T12) sub-lineage (mean pairwise distance of 4 (range: 0–9) core genome SNVs). This sub-lineage has been present in Western Africa and Middle Africa (Cameroon) since 2009 [9].

Most of our Wave 3 isolates (34/44) clustered in a discrete group of 69 isolates (mean pairwise distance of 7 (range: 0–24) core genome SNVs). This group was named Pre-AFR15 because it also contains a sub-lineage recently introduced into Eastern/Southern Africa previously named AFR15 by Smith et al. (magnification B in Figure 2) [19]. The European isolates collected in 2022 clustering in the Pre-AFR15 group were associated with travel to Iraq (n = 16), Pakistan (n = 8), Iran (n = 1), Afghanistan (n = 1), India (n = 1), Bangladesh (n = 1) and the United Arab Emirates (n = 1). One isolate was collected from a patient reporting no international travel and four isolates were obtained from patients whose travel history was unknown. Before the addition of our European isolates from 2022, this group contained mostly South Asian (2019–2022) isolates, one Iraqi isolate (2022) and six isolates collected in South Africa in 2023 (including three epidemiologically linked to the widespread cholera outbreak that occurred in Malawi) [19]. This group of strains harbours the *vprA* mutation (VC1320), which abolishes colistin resistance. The variant of ICE*Vch*Ind5 with a 10 kb deletion was present in three of the eight Pakistani isolates and all 16 Iraqi isolates.

Five isolates associated with Bangladesh belonged to the BD1.2 subclade [28]. Four of these isolates presented a disruption (insertion of IS*Vch4* between nt 327 and 328 of *wbeT*, GenBank accession number JF284685) of the *wbeT* (*rfbT*) gene suggestive of an Inaba serotype. Only one (22040725-VUM) of these four isolates was tested by serological slide agglutination; it was shown to belong to serotype Inaba.

## Discussion

In 2022, 472,697 cholera cases — more than double the 223,370 cases in 2021 — were reported to the WHO by 44 countries, accounting for 2,349 deaths (case fatality rate of 0.5%) [3]. However, these reported cases may not reflect the true morbidity of cholera because not all countries report cholera cases to the WHO, either through deliberate choice or due to low laboratory capacities. Furthermore, countries are only invited to notify the WHO of “any event occurring on its territory that may constitute a public health emergency of international concern”; the declaration of cholera cases is not mandatory [29]. This global upsurge of cholera also led to an increase in the number of imported cases in Europe, with 51 cases (vs only five in 2021) being reported, from which 49 *V. cholerae* O1 isolates were recovered. Due to the design of our microbiological study, with the use of very basic metadata, it was not possible to determine whether the patients infected abroad were holidaymakers, business travellers, humanitarian workers or persons linked to particular European countries by a common colonial and/or immigration history. Our sampling was based on only nine of the 53 countries covered by the WHO Regional Office for Europe (https://www.who.int/europe/about-us/about-who-europe), which reported few cholera cases in patients who had acquired the disease principally in Asian countries. Our findings are therefore unlikely to be representative of the full genetic diversity of the pathogen in 2022.

Our analysis confirmed that the 46 good-quality genomes belonged to the 7PET lineage. This confirmation is important because there are other lineages of *V. cholerae* O1, some even carrying CTX and toxin-coregulated pilus (TCP) genes, that are epidemiologically different from epidemic cholera (i.e. generally causing sporadic infections with rare secondary transmission events) [10]. One such non-7PET lineage is the *V. cholerae* O1 ST75 lineage isolated on the US Gulf Coast in the 1970s and now found in eastern Asia and South Africa [30].

Only two isolates belonged to an older 7PET group (Wave 2). For one of the cases, travel to the Philippines (a country that reported 8,098 cases in 2022) was reported. This 2022 isolate was phylogenetically related to three 2011 isolates from the Philippines (cholera outbreak in Palawan resulting in 500 notified cases and 20 deaths) [20]. At the time, the authors indicated that these three isolates were unique and belonged to an indigenous *V. cholerae* O1 population present locally in the aquatic ecosystem of the Philippines. Our comprehensive analysis shows that this strain circulated in the Philippines between 2011 and 2022 but was also found in South Korea in 2016, with closely related strains circulating in Eastern and South-eastern Asia and Melanesia since the 1990s.

All the other *V. cholerae* O1 isolates belonged to the most recent Wave 3 group (i.e. isolates carrying the *ctxB7* allele). Most (34/44) of the Wave 3 isolates were phylogenetically grouped into Pre-AFR15. This group probably made a major contribution to the global upsurge of cholera cases in 2022 as it was found in patients returning from countries that reported cases in 2022 but not in 2021 (such as Iraq, with 3,708 cases in 2022) or that reported a significant increase in the number of cases between 2021 and 2022 (Afghanistan, increase from 4,755 to 281,485; Pakistan, increase from 21 to 1,006). One Pre-AFR15 isolate was obtained from a patient returning from Iran, a country that did not report any cholera cases to the WHO in 2022. Interestingly, a recent study showed that the predominant strain in the cholera outbreak that occurred in Lebanon in 2022 (5,715 cases according to the WHO) also belonged to Pre-AFR15 and that cholera outbreaks in neighbouring countries, such as Syria in 2022 (70,222 cholera cases), might also have been caused by the same strain [27]. It has been shown that Wave 3 strains with the *ctxB7* allele, which was identified in Southern Asia in the early 2000s before spreading globally, reaching Haiti, in particular, in 2010, have a phenotypic profile compatible with hypervirulence (e.g. enhanced toxin and haemolysin production, hypermotility and greater fitness during colonisation and lethality in infant mice) [31]. The combination of such bacterial traits with a particular massive cholera outbreak in Southern Asia, the epicentre of the disease, such as the cholera outbreak exacerbated by flash floods in Pakistan in 2022 [32,33], might be sufficient to explain the successful spread of the AFR15 sub-lineage into the Middle East and Eastern Africa via infected humans travelling along trade routes. However, additional phenotypic studies are required to rule out the possibility that this currently emerging sub-lineage has become even more virulent than the earliest Wave 3 isolates carrying the *ctxB7* allele.

In 2022, African countries reported a total of 100,437 cases [3]. However, only five of our cases were associated with Africa, and all five were associated with a single country, Cameroon, which reported 14,431 cases in 2022.

Five isolates belonging to the BD1.2 subclade were associated with Bangladesh. Four of these isolates were assigned to serotype Inaba based on genomic analysis (this classification was confirmed by conventional serotyping for one of these strains). Interestingly, Monir et al. reported a massive cholera outbreak in Bangladesh in 2022 (the largest in 20–25 years) due to a strain belonging to subclade BD1.2 (displacing other subclades) with an Inaba serotype [28].

Antimicrobial resistance is an important evolutionary trait in the 7PET lineage [28]. By providing information about the AMR genes present in recent isolates from various parts of the world, our study improves knowledge about the recent evolution and antimicrobial susceptibility data of this pathogen of potential value for guiding the empirical treatment of cases infected in these regions of the world. No unusual extended AMR profiles were inferred from the analysis of the AMR gene content of the 46 genomes. Indeed, the two Wave 2 isolates had no AMR genes, and 31 of the 44 Wave 3 isolates — belonging to either AFR12 or Pre-AFR15 — had a ICE*Vch*Ind5 element with a deletion resulting in the loss of four AMR genes. The Pre-AFR15 and BD1.2 isolates had the *vprA* (VC1320) mutation associated with a reversal of susceptibility to polymyxins. However, there are recent reports, in Yemen in 2019, Zimbabwe in 2018 and Lebanon in 2022, of highly drug-resistant 7PET isolates belonging to the Wave 3 AFR13 sub-lineage [7,27,34]. These highly drug-resistant AFR13 isolates remained susceptible to only one antimicrobial recommended for cholera treatment: doxycycline in Yemen and Lebanon and azithromycin in Zimbabwe [7,27,34,35]. Indeed, these AFR13 isolates possessed QRDR mutations leading to quinolone resistance and also contained a large IncC plasmid carrying *bla*_PER-7_ (Yemen and Lebanon) or *bla*_CTX-M-15_ (Zimbabwe) genes encoding extended-spectrum beta-lactamases (ESBLs). This IncC plasmid can also carry the *mph(A), mph(E)* and *msr(E)* genes encoding resistance to azithromycin (as in Yemen and Lebanon) or *tet(A),* encoding intermediate or full resistance to tetracycline (Zimbabwe) [7,27,34]. It is therefore important to perform regular monitoring for AMR in 7PET *V. cholerae* O1 isolates.

## Conclusion

In conclusion, following the global upsurge of cholera cases in 2022, a ten-fold increase in the number of imported cholera cases in Europe was observed 2021–2022. In high-income countries in which cholera is not endemic, conventional laboratory determinations of *V. cholerae* O1, potentially complemented by the identification of *ctxA* or *ctxB* genes, are insufficient for formal assignment of the isolates to the 7PET lineage, the only lineage responsible for the current cholera pandemic and likely to trigger a public health response, depending on the context. In these high-income countries, we therefore strongly recommend the referral of all *V. cholerae* isolates to national reference centres for further genomic analyses. Phylogenomic analyses provide a sufficiently high resolution for robust surveillance, shedding light on the circulating strains and their evolution, particularly in terms of AMR. Furthermore, collaborative genomic studies should be performed regularly, in a timely manner, to improve our understanding of the global epidemiology of cholera.

## Supplementary Data

117000 Supplementary Material
